# Banking on the past: seed banks as a reservoir for rare and native species in restored vernal pools

**DOI:** 10.1093/aobpla/plt043

**Published:** 2013-09-25

**Authors:** Akasha M. Faist, Scott Ferrenberg, Sharon K. Collinge

**Affiliations:** 1 Department of Ecology and Evolutionary Biology, University of Colorado, Boulder, CO 80309, USA; 2 Environmental Studies Program, University of Colorado, Boulder, CO 80309, USA

**Keywords:** Annual plants, ephemeral wetlands, invasive species, native plants, rare species, seed bank, species diversity, vernal pools.

## Abstract

Soil seed banks serve as reservoirs for future plant communities, and when diverse and abundant can buffer vegetation communities against environmental fluctuations. Sparse seed banks, however, may lead to future declines of already rare species. Seed banks in wetland communities are often robust and can persist over long time periods making wetlands model systems for studying the spatial and temporal links between above- and belowground communities. Using collected soils and germination trials, we assessed species diversity and density in the seed banks of restored, ephemeral wetlands (vernal pools) in California's Central Valley, USA. Using long-term vegetation surveys, we compared the community structure of seed banks to that of aboveground vegetation and assessed the temporal links between below- and aboveground communities. We also compared the proportional abundances of different cover classes as well as the abundance of native plants in seed banks to aboveground communities. The proportional abundances of both rare and native species were greater in seed bank samples than in aboveground samples, yet the seed bank had lower species richness than aboveground vegetation. However, the seed bank had greater levels of differentiation among pools (beta diversity; β) than aboveground samples. Additionally, the seed bank was more similar to the earlier (2003–06) aboveground community than the more recent (2007–10) aboveground community. The correlation of species composition in the current seed bank to an earlier aboveground community suggests that seed banks exhibit storage effects while aboveground species composition in this system is not driven by seed bank composition, but is perhaps due to environmental filtering. We conclude that the seed bank of these pools is neither prone to the same temporal rates of invasion as the aboveground community, nor is seed abundance presently a limiting factor in the aboveground frequency of native species or a promoting factor in plant invasions of these restored habitats.

## Abstract (precis)

We found the belowground community in the soil seed bank of restored vernal pools has been less invaded by exotic plants and is a reservoir for rare and native plant species. We also found that seed bank community structure most closely resembled the aboveground community structure from five to eight years prior to seed bank sampling rather than more recent years. The maintenance of rare and native plant species in soil seed banks, even as aboveground vegetation communities become invaded by exotic plants is an exciting finding with important implications for management and restoration efforts in annual plant communities.

## Introduction

The existence of dormant, soil seed banks can allow different vegetation communities to occupy the same space at different periods of time (Chesson and Huntly 1997; Chesson 2000*a*, *b*; Chesson *et al.* 2004; Angert *et al.* 2009). Defining a vegetation community based on the active, aboveground species composition at a site can thus be incomplete, as there may be other species waiting to emerge following environmental or structural shifts (Warner and Chesson 1985). By allowing plants to store propagules belowground until adequate germination conditions are met, seed banks can buffer communities against perturbations (Templeton and Levin 1979; Thompson and Grime 1979; Leck 2001) and their composition can be a useful metric for estimating the *potential* future composition of the vegetation community (Major and Pyott 1966). Different rates of compositional turnover in belowground seed banks than in aboveground communities might lead to dramatic shifts in vegetation community structure following disturbances—shifts that could either enhance or limit native species abundance and diversity depending on seed bank assembly rates and processes.

Systems documented to build dense and diverse seed banks, such as wetlands, may be those that are disproportionately impacted by highly fluctuating environmental conditions over time (Brock and Rogers 1998; Facelli *et al.* 2005; Aponte *et al.* 2010). The changing availability of water in wetlands, for example, may cause plant propagules to lie dormant during moisture-poor or overly saturated conditions, and then germinate when their appropriate moisture regime is present (Cohen 1966; Templeton and Levin 1979; Venable and Brown 1988; Pake and Venable 1996; Bliss and Zedler 1997; Leck 2001). If the species- or community-specific environmental conditions for germination are not met, native species could be adversely impacted over time.

Our study focuses on vernal pools, which are ephemeral wetlands that are intermittently inundated over annually repeated cycles of flooding and drying in California's Mediterranean climate zone. Thus, vernal pools often experience greater variability in micro-climate than other wetland ecosystems. Also, vernal pools are populated almost exclusively by annual plants that are known to persist and propagate from seed (Keeley and Zedler 1998). The annual plants in this community (along with one common perennial species, *Eryngium vaseyi*) generally emerge during the wet-season's early rainfall and persist in immature states during periods of water inundation. During the relatively short, warm spring, the pools dry and these annual species quickly bolt to reach reproductive maturity and disperse their seeds before the onset of the hot dry summer (Linhart 1974; Keeley and Zedler 1998). The combination of highly variable water depths and inundation periods, as well as dominance by annual plants tends to promote a substantial seed bank in vernal pools, making them ideal models for studying seed bank structure and the links between seed banks and the aboveground vegetation community over time.

Vernal pools are found in Mediterranean climates worldwide, but are also present wherever strong seasonal precipitation patterns occur on flat or depressed surfaces that allow for prolonged inundation (Keeley and Zedler 1998; Deil 2005; Aponte *et al.* 2010). Globally, 50 % of wetland habitats have been lost (Zedler and Kercher 2005) and vernal pools in particular have been disproportionately affected with losses of over 90 % of their total area. Much of this habitat loss in vernal pools has been along the west coast of the USA, particularly in California's Central Valley, as a result of land development and agriculture (Zedler 2003; Zacharias and Zamparas 2010; Rhazi *et al.* 2012). Because of their highly variable and unique environmental conditions, vernal pools historically hosted many rare and endemic annual plant species (Croel and Kneitel 2011) that are now threatened due to habitat loss (Zedler 2003). Thus, the preservation and restoration of vernal pools is of great interest as they are critical habitats for the maintenance of biological diversity.

In addition to loss of habitat area, vernal pools have been negatively affected by invasive species―creating an additional threat to native biodiversity (Gerhardt and Collinge 2007; Collinge *et al.* 2011, 2013). Historically, California's vernal pools were assumed to be buffered from invasive species due to inherently variable environmental conditions―primarily annual flooding―which prevented the establishment of exotic plants less adapted to annual inundation (Gerhardt and Collinge 2003, 2007; Marty 2005; Price *et al.* 2010*b*). Nevertheless, vernal pools have experienced exotic species encroachment as invasive plant species have flourished across California's Mediterranean landscapes and as historic environmental buffers have weakened due to recent, shorter inundation times (Gerhardt and Collinge 2003, 2007; Collinge *et al.* 2011, 2013). Understanding how this recent influx of invasive species into the vernal pool system has altered the seed bank, and thus the *potential* of the community, is of great interest for the restoration and conservation of these threatened habitats.

To understand how exotic plant invasions might influence soil seed banks, and ultimately how they might influence the success of restored vernal pools, we compared the long-term records of aboveground plant communities to co-located samples of the belowground (seed bank) communities of a vernal-pool restoration site established in 1999 in California's Central Valley. We compared species composition and diversity, proportional abundances of rare, intermediate and common occurrence categories, and the proportional abundance of native and invasive species in both communities. Our primary goal was to understand how the aboveground vegetation compared with its seed bank counterpart belowground. Specifically, our three objectives were (i) to determine if the community structure of the seed bank matches that of the aboveground community, and if so, over what sampling period, (ii) to determine if the abundances of rare species in the aboveground community are constrained by their abundances in the seed bank, and (iii) to compare the abundances of invasive and native species in the aboveground versus seed bank community to understand how levels of invasion differ between the active and dormant communities.

## Methods

### Study site and experimental design

We conducted our study on the Travis Air Force Base (Solano County, CA, USA) (38°15′00″, 122°00′00″). The 15-ha site contains both naturally occurring, ‘reference’ and restored vernal pools with 256 experimental pools that were constructed in 1999 to mimic nearby reference pools (Collinge and Ray 2009; Collinge *et al.* 2013). Our original restoration experiment involved the establishment of a permanently marked, 50 cm × 50 cm square sampling plot in each of the 256 pools into which different seeding treatments were imposed, to track community trajectories within and among pools in relation to the initial seeding treatments. We have sampled plant species composition in these 0.25 m^2^ sampling plots annually since 2002, when all of the seeding treatments were completed. For the present study we selected a subset (*n* = 57) of permanent plots in restored pools evenly distributed among size classes (5 × 5, 5 × 10 and 5 × 20 m) and spatially dispersed across the entire restoration site.

Our research site experiences a Mediterranean climate, with a mean annual temperature of 20.1 °C and a mean annual rainfall of 500 mm, with over half of the precipitation falling between the months of November and February (Climate of Sacramento, Report 2010). The vernal pool hydroperiod closely follows the seasonal rain cycle; pools are filled by winter rains, followed by rapid spring drying, and a subsequent hot, dry summer and fall. Because vernal pools are fed by precipitation, they predominantly occur in areas with low to no-slope topography (Bauder 2005) and on soils with a high clay content that facilitates flooding through low soil infiltration (Rains *et al.* 2008).

### Seed bank sampling

We characterized the seed bank at each sampling location within a pool by collecting soil samples adjacent to permanently established vegetation sampling plots in restored pools (Collinge and Ray 2009) that had an observed peak inundation depth of 5–10 cm. We collected one soil sample from each of 57 sampling locations, comprised of a 125-cm^3^ cube from the soil surface to a 5 cm depth. We collected soil samples in March 2010 to coincide with the period when aboveground vegetation had reached peak germination, but prior to seed set, which ensures that samples were representative of the seed bank and not a measure of recently dispersed seeds. We air-dried samples in paper bags and then split them into equal parts by mass, storing one half of each sample for use in future studies.

### Seedling emergence

To determine seed abundance in the seed bank, we used a standard germination emergence method (Gross 1990; Bernhardt *et al.* 2008; Price *et al.* 2010*a*). In November 2010, we soaked the greenhouse-designated soil samples for 12–24 h to loosen the highly compact clay-rich soils and spread a thin layer (∼0.5 cm deep) of the mixture in round pots (7.6 cm diameter × 3.8 cm deep) with a base of potting soil (Fafard II, Fafard Inc., Agawam, MA, USA). We maintained soil moisture at saturation in each pot for ∼60 days, or until there were 14 consecutive days with no new germinants observed. We repeated this process three additional times with each soil sample, allowing soils to dry down for a minimum of 2 weeks between trials. We then counted germinants and identified individuals to species in each of the four trials. If we could not identify the species in the germinant phase, we allowed the individual to flower for identification purposes. We observed little to no mortality across germinated samples (A. M. Faist, unpubl. data) so we did not evaluate patterns of mortality in our analyses. We could not identify nine possible species in the seed bank observations; because of their low presence (accounting for 0.03 of the total counts) and to avoid inflation of diversity estimates, they were removed from the total species count and the rest of the analyses.

To accurately assess what seeds were found in the soil and because seed bank studies in different systems have used soil sorting as a common assessment tool (Gross 1990; Price *et al.* 2010*a*), we manually sorted seeds from the remaining half of each soil sample for community comparison to the emergence trials. We found that manual sorting did not reveal additional species not already present in greenhouse emergence trials, and also that the community identified by sorting did not significantly differ in species composition and abundances (PERMANOVA; *P* > 0.05) or diversity (Shannon *H*′ index; *P* > 0.05) from the community found in greenhouse emergence trials (A. M. Faist, unpubl*.* data). Because we found no evidence that manual sorting improved our characterization of the seed bank community, we included only the data from greenhouse emergence trials in the analyses presented here.

### Aboveground plant community

For the present study we used plant species composition data gathered annually (2003–10) from permanent plots, one plot per vernal pool, directly adjacent to where soil samples were extracted. For each aboveground plot (50 cm × 50 cm = 0.25 m^2^) we recorded the frequency of each plant species during peak flowering (April) using a 100-cell sampling grid (5 cm × 5 cm per cell). We divided the aboveground data into two temporal subsets: early aboveground (2003–06) and late aboveground (2007–10). The division of aboveground data between 2006 and 2007 was chosen (i) to create even-aged community cohorts, (ii) to account for an above-average precipitation spring (2005–06) that significantly altered the vegetation community after 2006 (see Collinge and Ray 2009; Collinge *et al.* 2011), and (iii) to facilitate testing of the paired relationship between aboveground community structure and soil seed bank community structure—i.e. the rapid change in the aboveground community provided us with a natural experiment to test for temporal links between the structure of above- and belowground communities.

### Data analysis

To assess whether our sampling intensity effectively captured species composition in our samples, we ran a species accumulation curve. Given the different scales of measure (frequencies versus counts) used for above- and belowground data collection, we chose not to use sample rarefaction methods (Simberloff 1972). Instead, we standardized aboveground vegetation frequency data and seed bank count data to proportional abundances for all analyses and examined curves relating species accumulation to sampling effort (sometimes considered as ‘individual rarefaction curves’)—a method used to assess the effectiveness of sampling effort for describing ecological communities (Gotelli and Colwell 2001). We then estimated the sampling efficiency of the three community groups from the ending slope (i.e. the slope of the line between the final two sample points) of each group's species accumulation curve.

We compared measures of alpha (α) diversity (species richness and Shannon *H*′) via Kruskal–Wallace tests followed by Steel–Dwass non-metric means comparisons in SAS-JMP 9.0.0 (JMP 2010). To avoid violating assumptions of sample independence, we analysed beta (β) diversity (measured here as Bray–Curtis dissimilarities) using ADONIS followed by the permutation method of ‘betadisper’ in the Vegan package for the R platform (Oksanen *et al.* 2011; Team RDC 2011).

To visualize the community structure of the aboveground vegetation groups and the seed bank we used non-metric multidimensional scaling (NMDS) ordinations using Bray–Curtis distance and 50 runs with real data, followed by 50 runs with randomized data in PC-ORD (MJM software; McCune and Mefford 2011). We compared the community structure of the three groups via PERMANOVA (a non-metric analysis that enables comparisons of species assemblages with an output similar to ANOVA) with 4999 randomizations in PC-ORD and with pairwise comparisons subjected to a Bonferroni sequential correction. To examine the relationships between the aboveground community sampled from 2007 to 2010 to the previous aboveground community from 2003 to 2006 and the seed bank community, as well as the relationship between the seed bank community and both aboveground communities, we used Mantel tests completed with PC-ORD. Mantel's *r* and *P* values (*P* = 1 + # of runs with Mantel *Z* ≥ observed *Z*/1 + # of randomized runs) were generated by PC-ORD with 5000 randomized runs.

We compared the proportional abundance of invasive and native vegetation, as well as the proportional abundances of rare, intermediately abundant and common species via *χ*^2^ tests. The rare category for the purpose of this study was defined as species that represented <2 % of the aboveground community's (2003–10) total abundance, intermediate species were those ≥2 to ≤10 % of aboveground abundance, and common species were those making up >10 % of total aboveground abundance.

## Results

### Species accumulation and diversity

**Figure 1.**
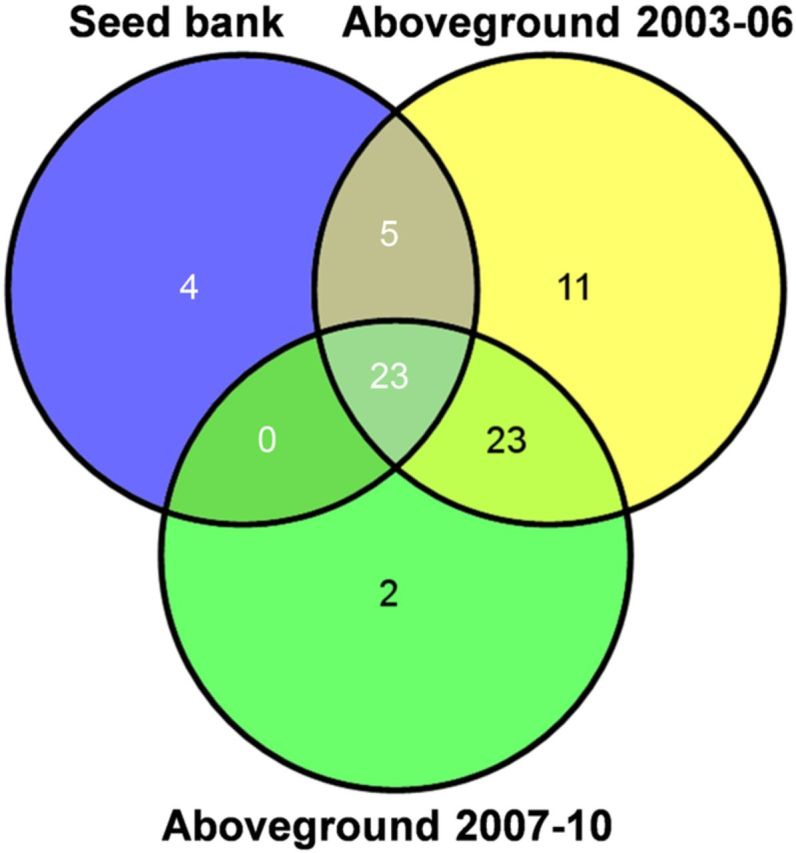
Plant species occurrence within and among seed bank samples (blue circle), and aboveground vegetation community samples from 2003–06 (yellow circle) and 2007–10 (green circle). Overlapping areas represent shared species; non-overlapping areas represent species unique to a community.

**Figure 2.**
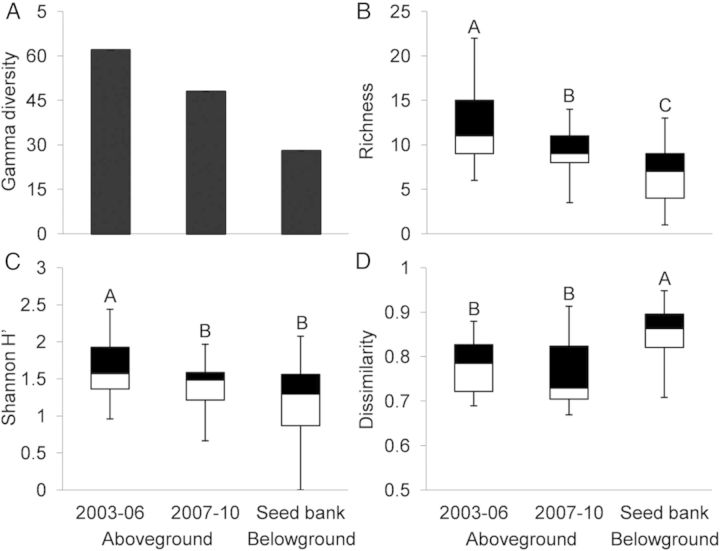
Above- and belowground species diversity measures from early (2003–06) and late (2007–10) aboveground vegetation communities, and from the soil seed bank: (A) gamma (landscape-level) diversity, (B) species richness, (C) Shannon–Weiner diversity index (*H*′) and (D) beta diversity measured as Bray–Curtis dissimilarity (a value of 1 indicates completely different communities, 0 indicates equivalent communities). The lettering above the box and whisker plots represents significant differences (*P* < 0.05) from sequentially corrected pairwise comparisons; error bars show 1.5IQR (inter-quartile range), black shading indicates the third quartile, white indicates the first quartile, and the dividing line shows the median.

**Figure 3.**
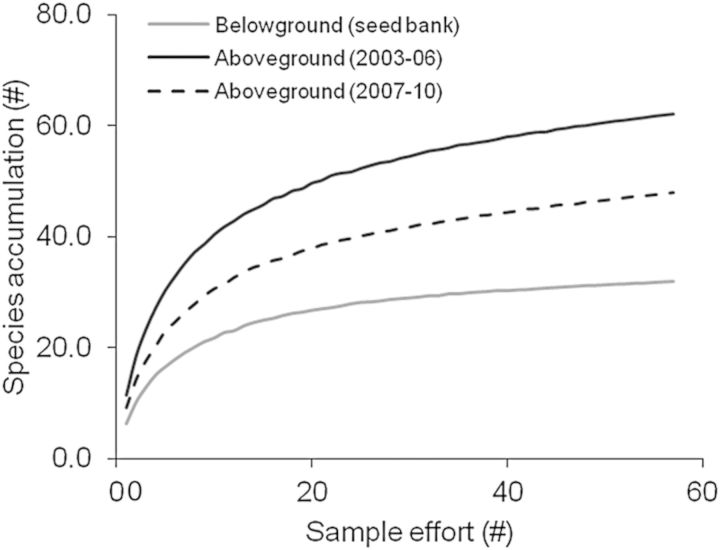
Species accumulation curves for early aboveground (2003–06), late aboveground (2007–10) and seed bank observations as a measure of sampling effort.

We found high seedling abundance from soil seed bank samples; emergence trials indicated that seed banks had a mean of 21 750 seeds m^−2^ of soil surface with a range from 1600 to 121 200 seeds m^−2^. Combining aboveground and seed bank communities, there were 68 plant species found among all sampled pools (Fig. 1). Landscape-level or gamma (γ) diversity in the early (2003–06) aboveground community was 62 species (Fig. 2A), while γ-diversity in the late (2007–10) aboveground community dropped to 48 species. Seed bank γ-diversity included 32 species. Species accumulation rates were similarly rapid across all three community groups (Fig. 3), with final slopes of the species accumulation curves indicating that overall sampling effort was more effective in the seed bank community than in the aboveground communities (Fig. 3). Specifically, finding an additional, unique species was estimated to require 10 additional samples (0.1 species per sample) in the seed bank community and 5 additional samples (0.2 species per sample) for both aboveground communities.

Sample-level or α-diversity measured both as species richness and as Shannon diversity (*H*′) was lower for the seed bank than for aboveground samples; the mean seed bank species richness of 6.4 (±0.32 SE) was significantly lower than that of aboveground vegetation in both the early and late sample periods (11.5 ± 0.54 and 9.1 ± 0.38, respectively) (*P* < 0.0001; Fig. 2B). The Shannon diversity (*H*′) of the seed bank (1.2 ± 0.06) was significantly lower than *H*′ in the early aboveground (1.6 ± 0.06) vegetation (*P* = 0.002; Fig. 2C), but was not significantly different from the late aboveground (1.4 ± 0.04) vegetation. β-Diversity (the amount of pairwise dissimilarity for samples) was significantly greater (*P* = 0.003; Fig. 2D) in the seed bank (0.85 ± 0.008) than in the early and late aboveground community (0.78 ± 0.008 and 0.75 ± 0.009, respectively), which did not differ significantly (*P* > 0.05).

### Community structure and species abundance

**Figure 4.**
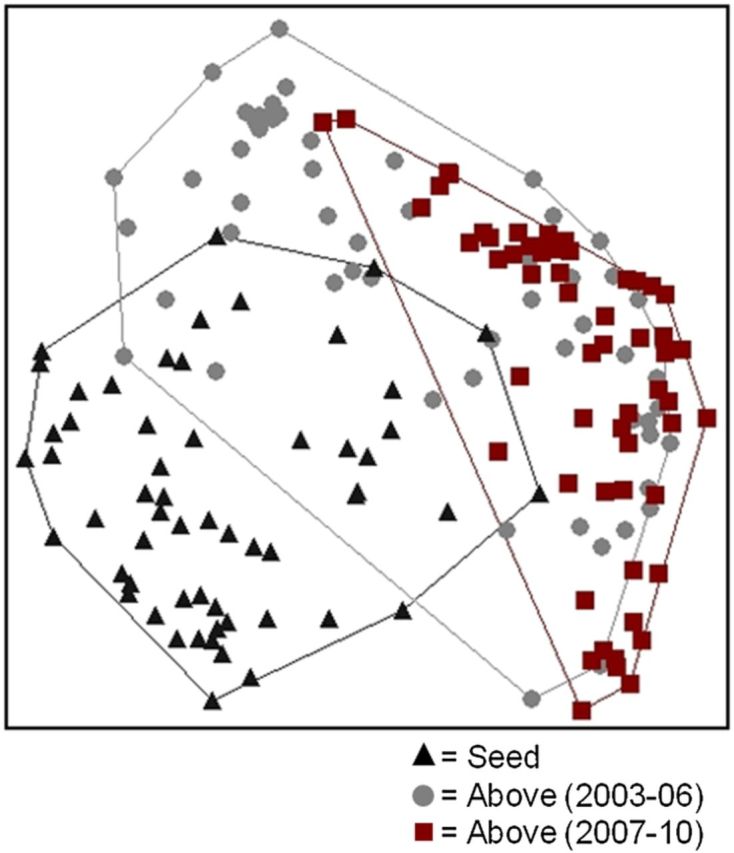
NMDS ordination comparing the within-sample, community composition of the seed bank (black triangles), the aboveground vegetation sampled from 2003 to 2006 (grey circles) and the aboveground vegetation sampled from 2007 to 2010 (red squares). NDMS axes = 4; final stress = 14.9.

The structure of the three community groups (seed bank, early and late aboveground) was significantly different (PERMANOVA *F*_2,168_ = 20.2, *P* < 0.001; Fig. 4). The results of our Mantel test showed a high similarity between the seed bank and the early aboveground observations (2003–06, *r* = 0.22, *P* = 0.0002) yet a low similarity with its more temporally related late aboveground (2007–10) observations (*r* = 0.11, *P* = 0.010) (Table 2). The early and late aboveground compared observations were the most closely related groups within these Mantel test results (*r* = 0.38, *P* = 0.0002).

**Figure 5.**
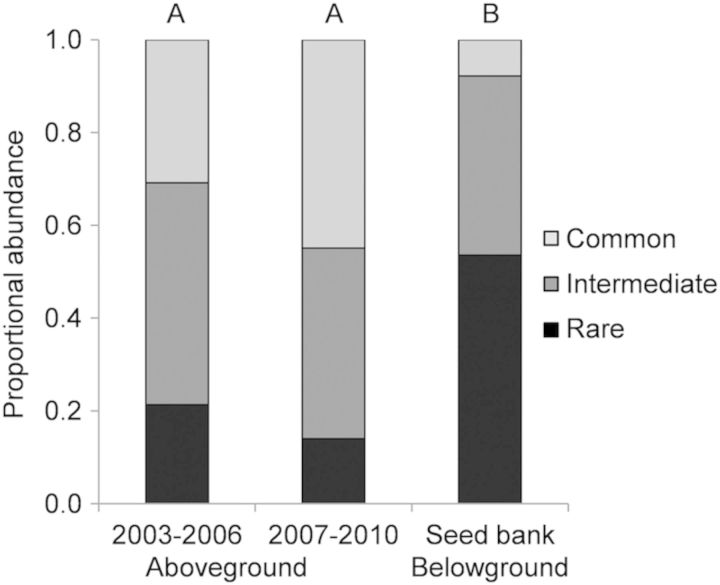
Proportion of the aboveground and seed bank vegetation community comprised of common, intermediate abundance and rare species. Dominance categories were determined by combined 2002–10 aboveground cover. Species with <2 % total cover were considered rare (*N* = 55 species), >2% and <10 % intermediate (*N* = 8 species) and if a cover of >10 % was found the species was considered common (*N* = 2 species). Letters above bars indicate significance differences from a *χ*^2^ test followed by Bonferroni sequential correction.

When the community was separated into dominance categories, we parsed 55 out of the 68 species in the aboveground community into the rare category (species representing ≤2 % of total abundance), while 8 species fell into the intermediate category (>2 to ≤10 % of total abundance) and 2 species were considered common (>10 % of total abundance). Proportional abundances of rare, intermediate and common species in the seed bank were significantly different from their abundances in both the early (2003–06) and late (2007–10) aboveground communities (*P* < 0.00001 for both comparisons; Fig. 5), while abundances for the two time periods of the aboveground community were not significantly different (*P* > 0.05). Combined rare species represented 21 and 14 % of the total community abundance in the aboveground samples (early and late, respectively), but represented a larger portion (54 %) of the seed bank community (Table 1; Fig. 5). Intermediate species represented 48 and 41 % of the early and late aboveground, respectively, and 39 % of the seed bank. Finally, the two common species made up 31 and 45 % of the early and late aboveground abundance but only 8 % of the seed bank.

**Table 1.** Percentage of the community comprised of native and introduced plant species categorized as rare (species combined), intermediate abundance and common in aboveground and seed bank vernal pool vegetation. ^a^Forb or graminoid. ^b^Perennial life form, all others annuals.

Abundance category	Aboveground		Seed bank	Functional type^a^	Native (Yes/No)
	(2003–06)	(2007–10)			
Rare (<2 % of total community; all combined)	21.34	13.98	53.58	na	na
Intermediate (≥2 to ≤10 %)					
*Lythrum hyssopifolium*	3.12	1.06	28.6	Forb	No
*Deschampsia danthonioides*	4.68	1.7	0	Gram	Yes
*Vicia villosa*	1.71	5.05	0	Forb	No
*Downingia concolor*	6.01	1.92	8.35	Forb	Yes
*Plagiobothrys stipitatus* (v. *micranthus*)	9.06	2.19	0.16	Forb	Yes
*Hordeum marinum*	4.15	12.01	0.35	Gram	No
*Lasthenia conjugens*	12.85	4.66	0.76	Forb	Yes
*Bromus hordeaceus*	6.19	12.42	0.4	Gram	No
Common (>10 %)					
*Eryngium vaseyi*^b^	8.46	16.07	2.82	Forb	Yes
*Lolium multiflorum*	22.41	28.86	4.97	Gram	No

The contributions of individual species to the relative abundances of the common and intermediate species categories were highly variable among the early and late aboveground and in the belowground seed bank communities (Table 1). In particular, the two most dominant species, *E. vaseyi* (native and the only perennial in the system) and *Lolium multiflorum* (invasive annual grass), were found in very high frequencies in the aboveground communities, but their belowground abundance was substantially lower (Table 1).

### Introduced and native species abundance

**Figure 6.**
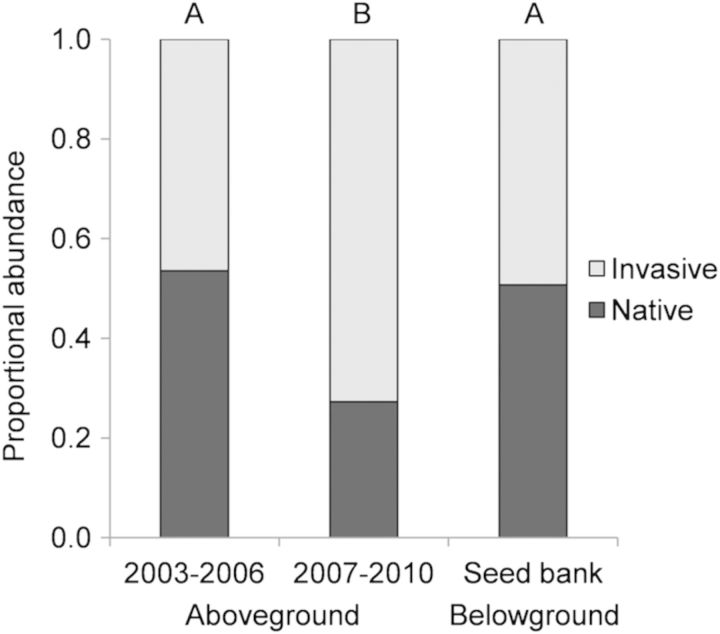
Proportion of the aboveground and seed bank vegetation community composed of native (black bar) and introduced (grey bar) species. Letters above bars indicate significant differences from a *χ*^2^ test followed by Bonferroni sequential correction.

The early (2003–06) and late (2007–10) aboveground communities had significantly different proportions of invasive and native species with a marked decrease in native species from the early to late aboveground community (*P* = 0.0002; Table 1, Fig. 6). The proportions of invasive and native species in the seed bank community were most similar to the early aboveground community, with no significant difference (*P* > 0.05) between the two groups, while the proportions of invasive and native species in the seed bank community were significantly different from those of the late aboveground community (*P* = 0.0007; Table 1, Fig. 6). Introduced species abundances represented 49 and 73 % of the early and late aboveground community, respectively, and 46 % of the seed bank composition. Natives represented 51 and 27 % of the overall abundances in early and late aboveground communities, respectively, and 54 % in the seed bank.

## Discussion

California's Central Valley, as with other Mediterranean climates, was historically the site of a vast network of seasonal, ephemeral wetlands (vernal pools) that have now largely been converted for agriculture and urban development. The remaining vernal pools harbour a large number of threatened native plants; thus there are active restoration efforts that address not only wetland habitat conservation and construction, but also the increasing levels of exotic plant invasions. In this aboveground versus belowground plant community study, our findings revealed that the aboveground abundances of rare and native plants in these restored vernal pools are not necessarily limited by their abundances in the belowground soil seed bank. Also, our results indicate that invasive plant encroachment in restored vernal pools has proceeded faster in the aboveground community than in the belowground seed bank community. This pattern of invasion is likely linked to a temporal disconnect in species composition between aboveground vegetation and the soil seed bank; indeed, we found that the community structure of the seed bank in these restored pools is more similar to aboveground plant community structure from 5–8 years prior (2003–06) than to aboveground community structure from only 1–4 years prior (2007–10). The higher proportional abundances of viable native and rare species in the soil seed bank than are currently found in aboveground vegetation suggest the potential for the community of these critical habitats to return to a more desired state―one replete with native plants and supportive of rare species―with changes in local environmental conditions.

### Species accumulation and diversity

Our results show that landscape-level (γ) diversity in the aboveground community appears to be decreasing over time (Fig. 2) possibly due to changes in seed banks. Mean sample-level (α) diversity was lowest in the seed bank, while sample-level dissimilarity (β-diversity) was highest in the seed bank (Table 1; Fig. 2). Lower α-diversity and greater β-diversity in seed bank samples suggest high spatial variation and species turnover belowground, possibly due to dispersal limitation or priority effects, or a combination of both, which have been shown to influence patterns of aboveground plant community assembly in these vernal pools (Collinge and Ray 2009; Collinge *et al.* 2013). Yet it is important to note that similar to measures of landscape γ-diversity, the median β-diversity of the aboveground community has decreased over time (though not yet significantly), suggesting a disparity between plants that germinate and emerge into the aboveground community and what is maintained in the seed bank. This pattern might be due to environmental filters (i.e. niche-based processes) strongly influencing the composition of aboveground communities in vernal pools, which could alter seed banks over time (e.g. Houle 1996; Benech-Arnold *et al.* 2000).

Importantly, our sampling effort does not appear to significantly influence measures of species diversity and dissimilarity in either the seed bank or the aboveground community characterizations. We found a similar, early rate of species accumulation in all three communities (Fig. 3), but with a decline toward an asymptotic value of species richness occurring more rapidly over the 57 independent samples in the seed bank than in the aboveground communities. This result is not surprising given the relatively low variation (i.e. standard errors of the mean values) in diversity measures across our study. Also, the low overall level of α-diversity in our study system, in conjunction with the high levels of β-diversity, indicate that a greater number of independent samples is likely preferable to an increase in each sample's size (either soil core volume or surface coverage aboveground), a result previously reported for other ecosystems dominated by annual plants with high seed densities like our system (e.g. Thompson 1986; Bigwood and Inouye 1988; Csontos 2007). Also, Bigwood and Inouye (1988) compared sampling methods for describing vegetation communities of ecosystems dominated by annual plants and characterized by high amounts of β-diversity over relatively small spatial scales, with their results leading them to recommend making seed bank samples smaller with a concomitant increase in sample number—an approach we attempted to employ.

### Community structure and species abundance

The differences observed at the community level between the seed bank and the aboveground categories were marked. The Mantel test (Table 2) illustrated that the seed bank similarity between the two aboveground groups (early and late) was not uniform. The higher seed bank similarity with the early aboveground data was initially surprising as there was a greater temporal lag between the two categories compared with the more recent 2007–10 aboveground seed bank comparisons. This temporal lag is discussed further in the following paragraphs, but an initial explanation could be that the seed bank received an influx of persistent seeds during the early observation period (2003–06) and as the aboveground community patterns changed—whether due to plant competition, environment or another variable (Collinge and Ray 2009; Collinge *et al.* 2011, 2013)—the seed bank has remained intact from the earlier years, causing a greater dissimilarity between the more recent aboveground monitoring periods.

**Table 2.** Mantel test for association between seed bank and aboveground vegetation communities. Mantel tests were completed on Bray–Curtis distance matrices; values of *r* and *P* are based on 5000 permutations.

Dependent matrix	Independent matrix	*r*	*P*
Seed bank	Aboveground (2003–06)	0.22	0.0002
Seed bank	Aboveground (2007–10)	0.11	0.010
Aboveground (2007–10)	Seed bank	0.11	0.009
Aboveground (2007–10)	Aboveground (2003–06)	0.38	0.0002

Further examining this above- and belowground dissimilarity, the aboveground dominance categories (Fig. 5, Table 1) display striking differences when compared with the seed bank. We found that these seed banks contained an abundance of vegetation considered rare in aboveground communities and lower frequencies of species that are highly common aboveground. Possible explanations for this disparity could be that the common aboveground species may have different seed bank strategies than the rare aboveground species. For instance, our sole perennial species (*E. vaseyi*) has the ability to return from established roots rather than relying on seeds to propagate. Also, some of the more common aboveground species may germinate nearly all of their seeds annually, depleting those species in the seed bank at the time of collection (Thompson *et al.* 1995). Inversely, species whose recruitment conditions are not met (i.e. rare species) may not germinate and are then maintained in the seed bank until appropriate conditions occur in the future (Leck and Simpson 1995). Other work in temporary ponds has found high seed densities and similar disparities between rare species abundance above- and belowground (Aponte *et al.* 2010). Together, these studies show that the definition of ‘rarity’ when only pertaining to aboveground presence can be misleading or quite different from seed bank ‘rarity’. Regardless, our results would seem to indicate that aboveground rarity is not determined by limitations in the seed bank, and that storage effects might promote future aboveground communities with higher abundances of ‘rare’ species.

### Introduced and native species abundance

In addition to our finding that the structure of the seed bank community more closely resembled the community structure of the early aboveground vegetation, we also found that the introduced-to-native species abundance ratios of the seed bank more closely resembled the early (2003–06) aboveground community rather than the late (2007–10, Fig. 6) one. Possible explanations for this pattern are that the native species have a higher longevity in the seed bank than the introduced species, allowing them to ‘wait out’ conditions that are not adequate for survival (Templeton and Levin 1979). Alternately, more natives in the seed bank might reflect limited germination rates of natives in response to competition with invasive annual plants—i.e. invasive plants may establish earlier in the season and out-compete natives which could deplete seed banks of invasives while maintaining natives (Tognetti and Chaneton 2012). Or lastly, because introduced species have only recently invaded these constructed vernal pools (Collinge *et al.* 2011), they may not have had sufficient time to build a substantial seed bank. In any case, introduced species have an ever-increasing presence in the aboveground community and now dominate each year's standing vegetation, yet they do not appear to be infiltrating the belowground community at similar rates and proportions.

## Conclusions

Our study revealed marked differences in aboveground vegetation and seed bank communities from restored vernal pools. We observed that the seed bank hosts a higher proportion of native and rare species, and has a community composition that more closely matches the composition of aboveground vegetation from 5–8 years prior than the composition from only 1–4 years prior. This discrepancy offers support for a ‘storage effect’ where different communities can occupy the same spatial location by being active versus dormant at different times. In addition, the relatively low representation of invasive species in the seed bank despite high aboveground frequencies suggests a legacy of native species maintaining populations in the seed bank. The seed bank composition, or *potential* of the community, indicates that this system could return to a native-dominated community with either an environmental shift or alternative management actions. Although many studies regarding restoration and invasion conclude with a sombre story, our research shows that the seed banks of restored vernal pools have not been overwhelmed with invasive species and that native species may once again thrive in these communities. The return of natives may occur in response to subtle environmental changes such as deepening the pools to create longer inundation periods or other management efforts geared toward the promotion of conditions favouring native-plant life histories.

## Sources of Funding

Our work was supported by the National Science Foundation (USA) Long Term Research in Environmental Biology grant (DEB-0744520), a National Science Foundation (USA) grant given to the greenhouse facility at the Department of Ecology and Evolutionary Biology (EBIO) at the University of Colorado (BIR-9317890) and the Society of Wetland Scientists (USA).

## Contributions by the Authors

All three authors have contributed substantially to this manuscript. The first author completed a majority of the writing, secured a portion of the funding and was involved in each step of the analyses. The second author completed a majority of the statistical analyses and was actively involved in writing the manuscript. The anchor author (third) was integral in securing funding for the bulk of this study, provided valuable knowledge of the study system, was responsible for the field data collection and aided in writing the manuscript.

## Conflicts of Interest Statement

None declared.
